# Non contiguous-finished genome sequence and description of *Clostridium jeddahense* sp. nov.

**DOI:** 10.4056/sigs.5571026

**Published:** 2014-05-01

**Authors:** Jean-Christophe Lagier, Fehmida Bibi, Dhamodharan Ramasamy, Esam I. Azhar, Catherine Robert, Muhammad Yasir, Asif A. Jiman-Fatani, Khalid Z. Alshali, Pierre-Edouard Fournier, Didier Raoult

**Affiliations:** 1 Unité de Recherche sur les Maladies Infectieuses et Tropicales Emergentes, UMR CNRS 7278 – IRD 198, Institut Hospitalo-Universitaire Méditerranée-Infection, Faculté de médecine, Aix-Marseille Université, France; 2 Special Infectious Agents Unit, King Fahd Medical Research Center, King Abdulaziz University, Jeddah, Saudi Arabia; 3 Department of Medical Laboratory Technology, Faculty of Applied Medical Sciences, King Abdulaziz University, Jeddah, Saudi Arabia; 4 Department of Medical Microbiology and Parasitology, Faculty of Medicine, King; Abdulaziz University, Jeddah, Saudi Arabia; 5 Department of Medicine, Faculty of Medicine, King Abdulaziz University, PO Box 80215, Jeddah, Postal code 21589, Saudi Arabia

**Keywords:** *Clostridium jeddahense*, genome, culturomics, taxonogenomics

## Abstract

*Clostridium jeddahense* strain JCD^T^ (= CSUR P693 = DSM 27834) is the type strain of *C. jeddahense* sp. nov. This strain, whose genome is described here, was isolated from the fecal flora of an obese 24 year-old Saudian male (BMI=52 kg/m^2^). *Clostridium jeddahense* strain JCD^T^ is an obligate Gram-positive bacillus. Here we describe the features of this organism, together with the complete genome sequence and annotation. The 3,613,503 bp long genome (1 chromosome, no plasmid) exhibits a G+C content of 51.95% and contains 3,462 protein-coding and 53 RNA genes, including 4 rRNA genes.

## Introduction

*Clostridium jeddahense* strain JCD^T^ (=CSUR P693 = DSM 27834), is the type strain of *Clostridium jeddahense* sp. nov. This bacterium is a Gram-positive, anaerobic, spore-forming indole, positive bacillus that was isolated from the stool of an obese 24 year-old Saudian individual, as a part of a culturomics study as previously reported.

The usual parameters used to delineate a bacterial species include 16S rDNA sequence identity and phylogeny [1,2], genomic G + C content diversity, and DNA–DNA hybridization (DDH) [3,4]. Nevertheless, some limitations appeared notably because the cutoff values vary dramatically between species and genera [5]. The introduction of high-throughput sequencing techniques made genomic data for many bacterial species available [6]. We recently proposed a new method (taxono-genomics), which includes genomic data in a polyphasic approach to describe new bacterial species [6]. This strategy combines phenotypic characteristics, including MALDI-TOF MS spectrum, and genomic analysis [7-37].

Here, we present a summary classification and a set of features for *C. jeddahense* sp. nov. strain JCD^T^ (=CSUR P693 = DSM 27834), together with the description of the complete genome sequencing and annotation. These characteristics support the circumscription of the species *C. jeddahense.*

The genus *Clostridium* was created in 1880 [38] and consists of obligate anaerobic rod-shaped bacilli able to produce endospores [38]. More than 200 species have been described to date (http://www.bacterio.cict.fr/c/clostridium.html). Members of the genus *Clostridium* are mostly environmental bacteria or associated with the commensal digestive flora of mammals. However, several are major human pathogens, including *C. botulinum*, *C. difficile* and *C. tetani* [38].

## Classification and features

**Figure 1:**
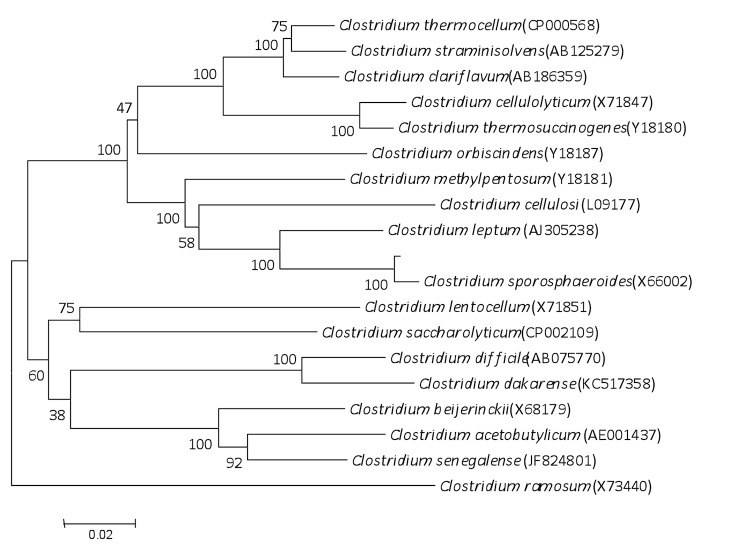
A consensus phylogenetic tree highlighting the position of *Clostridium jeddahense* strain JCD^T^ relative to other type strains within the *Clostridum* genus. GenBank accession numbers are indicated in parentheses. Sequences were aligned using CLUSTALW, and phylogenetic inferences were obtained using the maximum-likelihood method in the MEGA software package. Numbers at the nodes are the percentages of bootstrap values from 500 replicates that support the node. *Clostridium ramosum* was used as outgroup. The scale bar represents a 2% nucleotide sequence divergence.

A stool sample was collected from an obese 24-year-old male Saudian volunteer patient living in Jeddah. The patient gave an informed and signed consent, and the agreement of the Ethical Committee of the King Abdulaziz University, King Fahd medical Research Centre, Saudi Arabia, and the local ethics committee of the IFR48 (Marseille, France) were obtained under agreement number 014-CEGMR-2-ETH-P and 09-022 respectively. The fecal specimen was preserved at -80°C after collection and sent to Marseille. Strain JCD^T^ (Table 1) was isolated in July 2013 by anaerobic cultivation on 5% sheep blood-enriched Columbia agar (BioMerieux, Marcy l’Etoile, France) after a 5-day preincubation on blood culture bottle with rumen fluid. This strain exhibited a 97.3% nucleotide sequence similarity with *Clostridium sporosphaeroides* strain DSM 1294 (Figure 1). This value was lower than the 98.7% 16S rRNA gene sequence similarity threshold recommended by Stackebrandt and Ebers to delineate a new species without carrying out DNA-DNA hybridization [2] and was in the 78. 4 to 98.9% range of 16S rRNA identity values observed among 41 *Clostridium* species with validly published names [52].

**Table 1:** Classification and general features of *Clostridium jeddahense* strain JCD^T^ according to the MIGS recommendations [39]

**MIGS ID**	**Property**	**Term**	**Evidence code^a^**
	Current classification	Domain *Bacteria*	TAS [40]
		Phylum *Firmicutes*	TAS [41-43]
		Class *Clostridia*	TAS [44,45]
		Order *Clostridiales*	TAS [46,47]
		Family *Clostridiaceae*	TAS [46,48]
		Genus *Clostridium*	IDA [46,49,50]
		Species *Clostridium jeddahense*	IDA
		Type strain JCD^T^	IDA
	Gram stain	Positive	IDA
	Cell shape	Rod	IDA
	Motility	Motile	IDA
	Sporulation	Sporulating	IDA
	Temperature range	Mesophile	IDA
	Optimum temperature	37°C	IDA
MIGS-6.3	Salinity	Unknown	IDA
MIGS-22	Oxygen requirement	Anaerobic	IDA
	Carbon source	Unknown	IDA
	Energy source	Unknown	IDA
MIGS-6	Habitat	Human gut	IDA
MIGS-15	Biotic relationship	Free living	IDA
MIGS-14	Pathogenicity Biosafety level Isolation	Unknown 2 Human feces	
MIGS-4	Geographic location	Jeddah, Saudi Arabia	IDA
MIGS-5	Sample collection time	July 2013	IDA
MIGS-4.1	Latitude	21.422487	IDA
MIGS-4.1	Longitude	39.856184	IDA
MIGS-4.3	Depth	Surface	IDA
MIGS-4.4	Altitude	0 m above sea level	IDA

Evidence codes - IDA: Inferred from Direct Assay; TAS: Traceable Author Statement (i.e., a direct report exists in the literature); NAS: Non-traceable Author Statement (i.e., not directly observed for the living, isolated sample, but based on a generally accepted property for the species, or anecdotal evidence). These evidence codes are from the Gene Ontology project [51]. If the evidence is IDA, then the property was directly observed for a live isolate by one of the authors or an expert mentioned in the acknowledgements.

**Figure 2:**
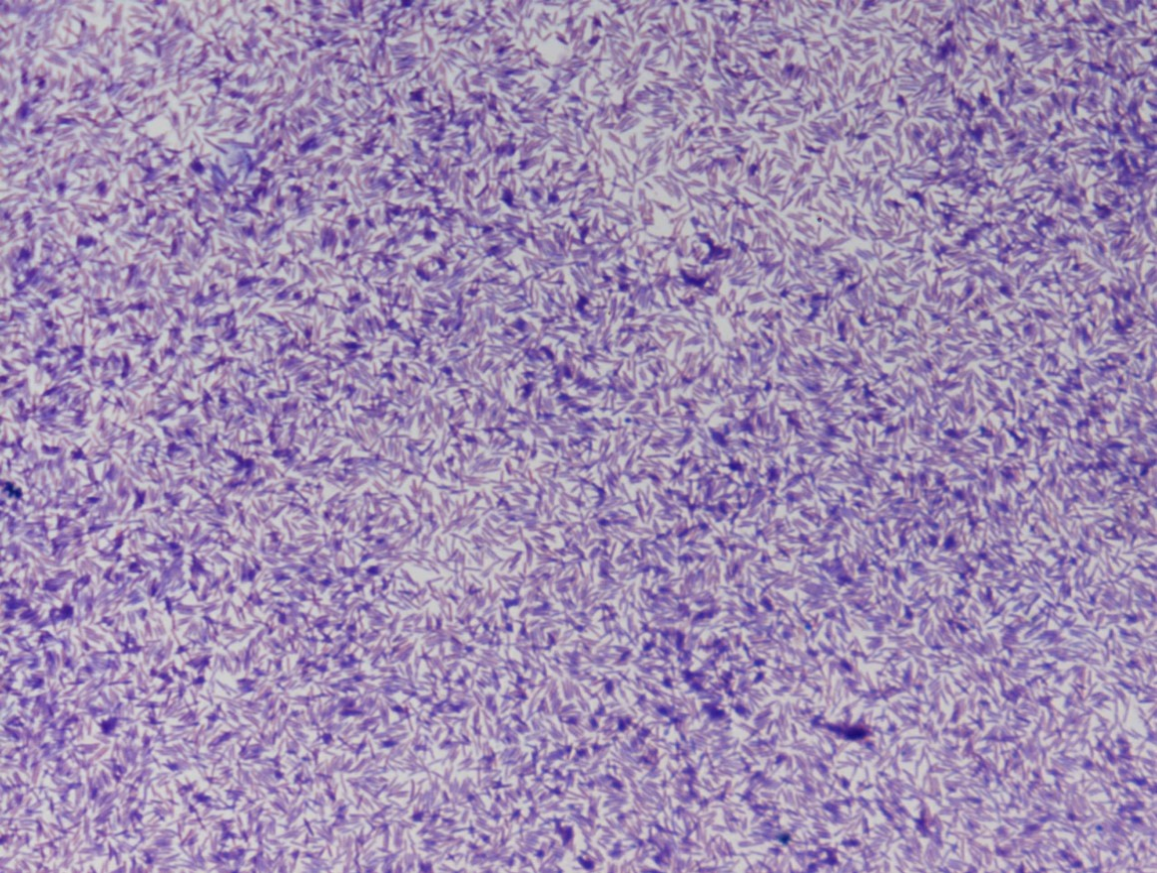
Gram stain of *Clostridium jeddahense* strain JCD^T^

**Figure 3:**
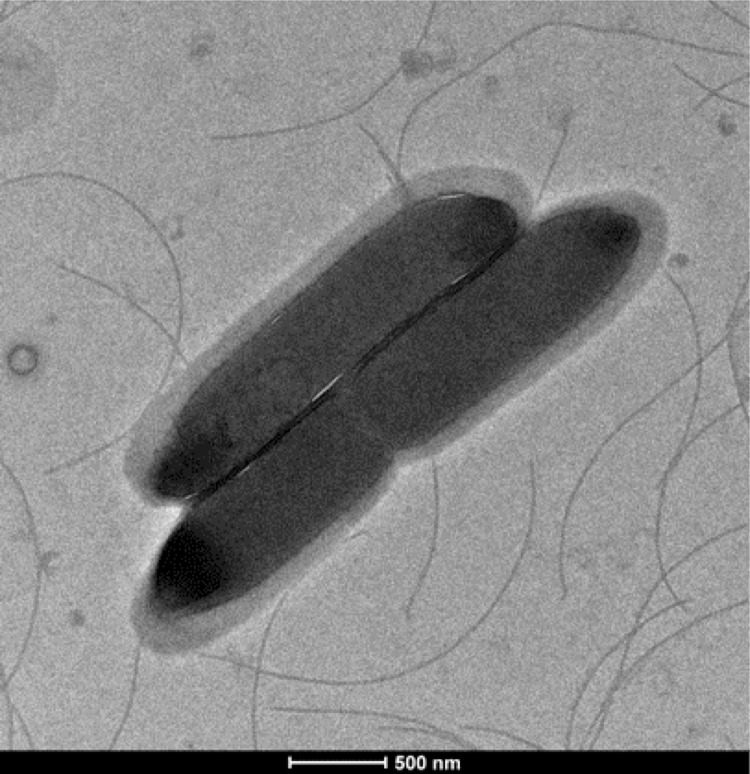
Transmission electron micrograph of *C. jeddahense* strain JCD^T^, taken using a Morgani 268D (Philips) at an operating voltage of 60kV.The scale bar represents 500 nm.

Four growth temperatures (25, 30, 37, 45°C) were tested; growth occurred between 25 and 37°C, but optimal growth was observed at 37°C, 24 hours after inoculation. No growth occurred at 45°C. Colonies were translucent and approximately 0.2 to 0.3 mm in diameter on 5% sheep blood-enriched Columbia agar (BioMerieux). Growth of the strain was tested on the same agar under anaerobic and microaerophilic conditions using GENbag anaer and GENbag microaer systems, respectively (BioMerieux), and in aerobic conditions, with or without 5% CO_2_. Growth was observed only anaerobically. No growth occurred in aerobic or microaerophilic conditions. Gram staining showed Gram-positive rods able to form spores (Figure 2). A motility test was positive. Cells grown on agar exhibit a mean diameter of 1 µm and a mean length of 1.22 µm in electron microscopy (Figure 3).

Strain JCD^T^ exhibited neither catalase nor oxidase activity (Table 2). Using an API Rapid ID 32A strip (BioMerieux), positive reactions were obtained for indole production, alkaline phosphatase, arginine arylamidase, proline arylamidase, alanine arylamidase, glycine arylamidase, histidine arylamidase, glutamyl glutamic acid arylamidase and serine arylamidase. Negative reactions were obtained for arginine dihydrolase, α-galactosidase, β-galactosidase, α-glucosidase, β-glucosidase, α-arabinosidase, N-acetyl-β-glucosaminidase, glutamic acid decarboxylase, α-fucosidase, nitrate reduction, leucyl glycine arylamidase, fermentation of mannose and raffinose, urease, β-galactosidase-6-phosphatase, β-glucuronidase, phenylalanine arylamidase, leucine arylamidase, pyroglutamic acid arylamidase and tyrosine arylamidase. Using an API 50CH strip (Biomerieux), strain JCD^T^ was asaccharolytic.

**Table 2:** Differential characteristics of *Clostridium jeddahense* JCD^T^, *C. senegalense* JC122 [11], *C. dakarense* FF1 [34], *C. beijerinckii* NCIMB 8052, *C. difficile* B1, *C. cellulolyticum* H10, *C. leptum* DSM 753 and *C. sporosphaeroides* DSM 1294 [53]†.

**Properties**	*C.* *jeddahense*	*C.* *sporosphaeroides*	*C.* *cellulolyticum*	*C.* *leptum*	*C.* *senegalense*	*C.* *dakarense*	*C.* *beijerinckii*	*C.* *difficile*
Cell diameter (µm)	1.0	0.5-0.6	1.5	0.6-0.8	1.1	1.2	1.7	3.0
Oxygen requirement	Strictly anaerobic	Strictly anaerobic	Strictly anaerobic	Strictly anaerobic	Strictly anaerobic	Strictly anaerobic	Strictly anaerobic	Strictly anaerobic
Gram stain	Positive	Positive	Positive	Positive	Positive	Positive	Variable	Variable
Motility	Motile	Non Motile	Motile	Non Motile	Motile	Motile	Motile	Motile
Endospore formation	+	+	+	+	+	+	+	+
Indole	+	-	-	Na	-	+	Na	Na
**Production of**								
Alkaline phosphatase	+	Na	Na	Na	-	+	Na	Na
Catalase	-	Na	-	Na	-	-	-	Na
Oxidase	-	Na	Na	Na	-	-	Na	Na
Nitrate reductase	-	-	-	Na	-	-	-	-
Urease	-	-	-	Na	-	-	-	Na
β-galactosidase	-	Na	Na	Na	-	-	Na	Na
N-acetyl-glucosamine	-	Na	Na	Na	+		Na	Na
**Acid from**								
L-Arabinose	-	-	Na	Na	Na	-	+	-
Ribose	-	-	Na	W	Na	-	-	-
Mannose	-	-	Na	-	Na	-	+	+
Mannitol	-	-	Na	-	Na	-	+	+
Sucrose	-	-Na	Na	W	Na	-	+	+
D-glucose	-	Na	Na	Na	Na	+	+	Na
D-fructose	-	Na	Na	Na	Na	-	+	+
D-maltose	-	Na	Na	Na	Na	+	+	-
D-lactose	-	Na	Na	Na	Na	-	+	-
**G+C content (%)**	52		41	51	26.8	27.98	28	28
**Habitat**	Human gut	Environment	Compost	Human gut	Human gut	Human gut	Human gut	Human gut

^†^(Na = data not available; w = weak, v = variable reaction)

*C. jeddahense* is susceptible to amoxicillin, amoxicillin-clavulanate, imipenem, metronidazole, doxycycline, rifampicin, vancomycin but resistant to ceftriaxone, ciprofloxacin and trimethoprim-sulfamethoxazole. The comparisons with other *Clostridium* species are summarized in Table 2.

**Figure 4:**
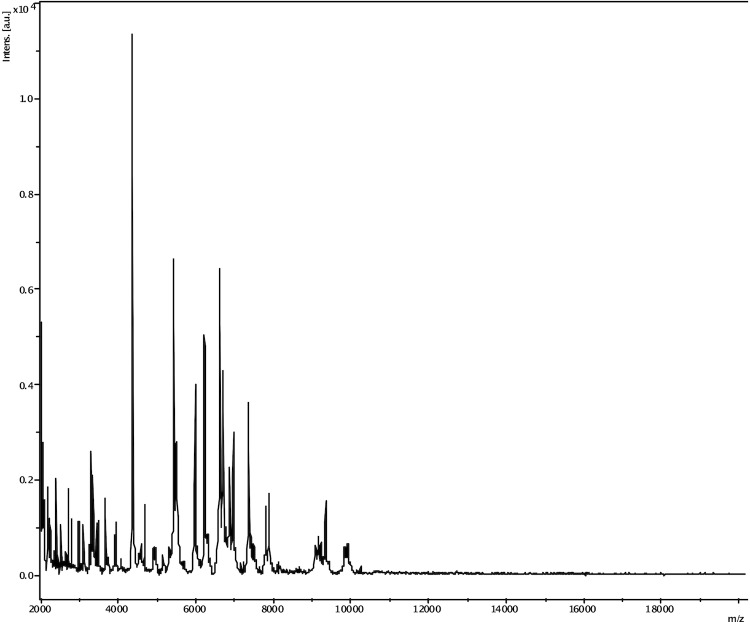
Reference mass spectrum from *C. jeddahense* strain JCD^T^. Spectra from 12 individual colonies were compared and a reference spectrum was generated.

**Figure 5:**
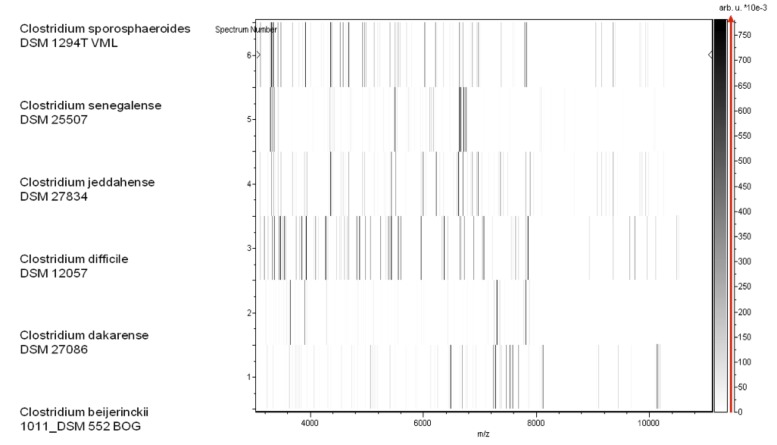
Gel view comparing *C. jeddahense* strain JCD^T^ to other *Clostridium* species. The gel view displays the raw spectra of loaded spectrum files arranged as a pseudo-electrophoretic gel. The x-axis records the m/z value. The left y-axis displays the running spectrum number originating from subsequent spectra loading. The peak intensity is expressed by a grey scale scheme code. The grey scale bar on the right y-axis indicates the relation between the shade of grey a peak is displayed with and the peak intensity in arbitrary units. Species names are shown on the left.

Matrix-assisted laser-desorption/ionization time-of-flight (MALDI-TOF) MS protein analysis was carried out as previously described [54]. Briefly, a pipette tip was used to pick one isolated bacterial colony from a culture agar plate and spread it as a thin film on a MTP 384 MALDI-TOF target plate (Bruker Daltonics, Leipzig, Germany). Twelve distinct deposits from twelve isolated colonies were performed for strain JCD^T^. Each smear was overlaid with 2 µL of matrix solution (saturated solution of alpha-cyano-4-hydroxycinnamic acid) in 50% acetonitrile, 2.5% tri-fluoracetic acid, and allowed to dry for 5 minutes. Measurements were performed with a Microflex spectrometer (Bruker). Spectra were recorded in the positive linear mode for the mass range of 2,000 to 20,000 Da (parameter settings: ion source 1 (ISI), 20kV; IS2, 18.5 kV; lens, 7 kV). A spectrum was obtained after 675 shots with variable laser power. The time of acquisition was between 30 seconds and 1 minute per spot. The twelve JCD^T^ spectra were imported into the MALDI BioTyper software (version 2.0, Bruker) and analyzed by standard pattern matching (with default parameter settings) against the main spectra of 3,769 bacteria, including 228 spectra from 96 *Clostridium* species. The method of identification included the m/z from 3,000 to 15,000 Da. For every spectrum, a maximum of 100 peaks were compared with spectra in database. The resulting score enabled the identification of tested species, or not: a score ≥ 2 with a validly published species enabled identification at the species level, a score ≥ 1.7 but < 2 enabled identification at the genus level, and a score < 1.7 did not enable any identification. No significant MALDI-TOF score was obtained for strain JCD^T^ against the Bruker database, suggesting that our isolate was not a member of a known species. We added the spectrum from strain JCD^T^ to our database (Figure 4). Finally, the gel view showed the spectral differences with other members of the genus *Clostridium* (Figure 5).

## Genome sequencing information

### Genome project history

The organism was selected for sequencing on the basis of its phylogenetic position and 16S rDNA similarity to members of the genus *Clostridium*, and is part of a study of the human digestive flora aiming at isolating all bacterial species in human feces [55]. It was the 101^st^ genome of a *Clostridium* species and the first genome of *C. jeddahense* sp. nov. The GenBank accession number is CBYL00000000. The assembly consists of 104 contigs. Table 3 shows the project information and its association with MIGS version 2.0 compliance [39].

**Table 3:** Project information

**MIGS ID**	**Property**	**Term**
MIGS-31	Finishing quality	High-quality draft
MIGS-28	Libraries used	Paired end and Mate pair
MIGS-29	Sequencing platform	MySeq Illumina
MIGS-31.2	Fold coverage	94.91×
MIGS-30	Assemblers	Newbler
MIGS-32	Gene calling method	PRODIGAL
	Genbank Date of Release	February 12, 2014
	Genbank project ID	CBYL00000000
MIGS-13	Project relevance	Study of the human gut microbiome

### Growth conditions and DNA isolation

*C. jeddahense* sp. nov., strain JCD^T^ (= CSUR P693 = DSM 27834) was grown on 5% sheep blood-enriched Columbia agar (BioMerieux) at 37°C in anaerobic atmosphere. Bacteria grown on three Petri dishes were harvested and resuspended in 4x100µL of TE buffer. Then, 200 µL of this suspension was diluted in 1ml TE buffer for lysis treatment that included a 30- minute incubation with 2.5 µg/µL lysozyme at 37°C, followed by an overnight incubation with 20 µg/µL proteinase K at 37°C. Extracted DNA was then purified using 3 successive phenol-chloroform extractions and ethanol precipitation at -20°C overnight. After centrifugation, the DNA was resuspended in 160 µL TE buffer.

### Genome sequencing and assembly

Genomic DNA of *Clostridium jeddahense* was sequenced on a MiSeq sequencer (Illumina, Inc, San Diego CA 92121, USA) with 2 applications: paired end and mate pair. The paired end and the mate pair strategies were barcoded in order to be mixed respectively with 14 other genomic projects constructed according the Nextera XT library kit (Illumina) and 11 others projects with the nextera Mate pair kit (Illumina).

The gDNA was quantified by a Qubit assay with the high sensitivity kit (Life technologies, Carlsbad, CA, USA) to 11.1 ng/µL and dilution was performed such that 1ng of each strain’s gDNA was used to construct the paired end library. The “tagmentation” step fragmented and tagged the DNA .Then limited cycle PCR amplification completed the tag adapters and introduced dual-index barcodes. After purification on Ampure beads (Life Technolgies, Carlsbad, CA, USA), the libraries were normalized on specific beads according to the Nextera XT protocol (Illumina). Normalized libraries are pooled into a single library for sequencing on the MiSeq. The pooled single strand library was loaded onto the reagent cartridge and then onto the instrument along with the flow cell. Automated cluster generation and paired-end sequencing with dual index reads was performed in a single 39-hour run at a 2x250 bp read length. Within this pooled run, the index representation was determined to be 7.3%. Total information of 5.3 Gbases was obtained from a 574 K/mm2 density with 95.4% (11,188,000 clusters) of the clusters passing quality control (QC) filters. From the genome sequencing process, the 753,292 produced Illumina reads for *Clostridium jeddahense* were filtered according to the read qualities.

The mate pair library was constructed from 1 µg of genomic DNA using the Nextera Mate Pair Illumina guide. The genomic DNA sample is simultaneously fragmented and tagged with a mate pair junction adapter. The profile of the fragmentation was validated on an Agilent 2100 BioAnalyzer (Agilent Technologies, Inc., Santa Clara, CA, USA) with a DNA7500 labchip. The DNA fragments range in size from 1 kb up to 11 kb with a mean size of 7kb. No size selection was performed and 600 ng tagmented fragments were circularized. The larger circularized DNA molecules were physically sheared to smaller sized fragments with a mean size of 625 bp on the Covaris device S2 in microtubes (Woburn, MA, USA) .The library’s profile and the quantitation were visualized on a High Sensitivity Bioanalyzer LabChip. The libraries were normalized to 2 nM and pooled. After a denaturation step and dilution at 10 pM the pool of libraries was loaded onto the reagent cartridge and then onto the instrument along with the flow cell. Automated cluster generation and sequencing run was performed in a single 39-hour run at a 2x250 bp read length.

Total information of 3.9 Gb was obtained from a 399 K/mm2 density with 97.9% (7,840,000 clusters) of the clusters passing quality control (QC) filters. Within this pooled run, the index representation for *Clostridium jeddahense* was determined to be 6.54%.

From this genome sequencing process, the 501,426 produced Illumina reads for *Clostridium jeddahense* were filtered according to the read qualities.

### Genome annotation

Open Reading Frames (ORFs) were predicted using Prodigal [56] with default parameters. However, the predicted ORFs were excluded if they spanned a sequencing gap region. The predicted bacterial protein sequences were searched against the GenBank [57] and Clusters of Orthologous Groups (COG) databases using BLASTP. The tRNAs and rRNAs were predicted using the tRNAScan-SE [58] and RNAmmer [59] tools, respectively. Signal peptides and numbers of transmembrane helices were predicted using SignalP [60] and TMHMM [61], respectively. Mobile genetic elements were predicted using PHAST [62] and RAST [63]. ORFans were identified if their BLASTP *E*-value was lower than 1e-03 for alignment length greater than 80 amino acids. If alignment lengths were smaller than 80 amino acids, we used an *E*-value of 1e-05. Such parameter thresholds have already been used in previous work to define ORFans. Artemis [64] and DNA Plotter [65] were used for data management and visualization of genomic features, respectively. The Mauve alignment tool (version 2.3.1) was used for multiple genomic sequence alignment [66].

 To estimate the mean level of nucleotide sequence similarity at the genome level between *C. jeddahense* and 7 other members of the genus *Clostridium*, we used the Average Genomic Identity Of gene Sequences (AGIOS) home-made software [6]. Briefly, this software combines the Proteinortho software [67] for detecting orthologous proteins between pairs of genomes, then retrieves the corresponding genes and determines the mean percentage of nucleotide sequence identity among orthologous ORFs using the Needleman-Wunsch global alignment algorithm. *C. jeddahense* strain JCD^T^ was compared to C. senegalense** strain JC122, *C. dakarense* strain FF1, *Clostridium beijerinckii* strain NCIMB 8052, *C. difficile* strain B1, *Clostridium cellulolyticum* strain H10, *Clostridium leptum* strain DSM 753, and *Clostridium sporosphaeroides* strain DSM 1294 (see Table 6B).

**Table 6B:** Genomic comparison of *C. jeddahense* with 7 other *Clostridium* species†

	*C.* *jeddahense*	*C.* *sporosphaeroides*	*C.* *cellulolyticum*	*C.* *dakarense*	*C.* *difficile*	*C.* *leptum*	*C.* *senegalense*	*C.* *beijerincki*
*C. jeddahense*	**3,462**	1,573	876	816	847	1,030	770	1,044
*C. sporosphaeroides*	91.97	**2,951**	854	776	819	1,016	745	1,015
*C. cellulolyticum*	61.60	60.62	**3,923**	806	851	754	814	946
*C. dakarense*	57.30	56.34	65.70	**5,020**	1,271	665	1,110	1,142
*C. difficile*	57.56	56.80	65.65	77.74	**3,390**	714	1,098	1,171
*C. leptum*	67.98	68.07	61.94	58.55	58.84	**3,591**	651	780
*C. senegalense*	57.52	56.73	65.71	70.18	69.41	58.72	**3,704**	1,125
*C. beijerincki*	58.63	57.95	65.93	68.98	68.48	59.58	71.37	**3,818**

†Numbers of orthologous proteins shared between genomes (above diagonal), AGIOS values (below diagonal) and numbers of proteins per genome (bold numbers)

## Genome properties

**Figure 6:**
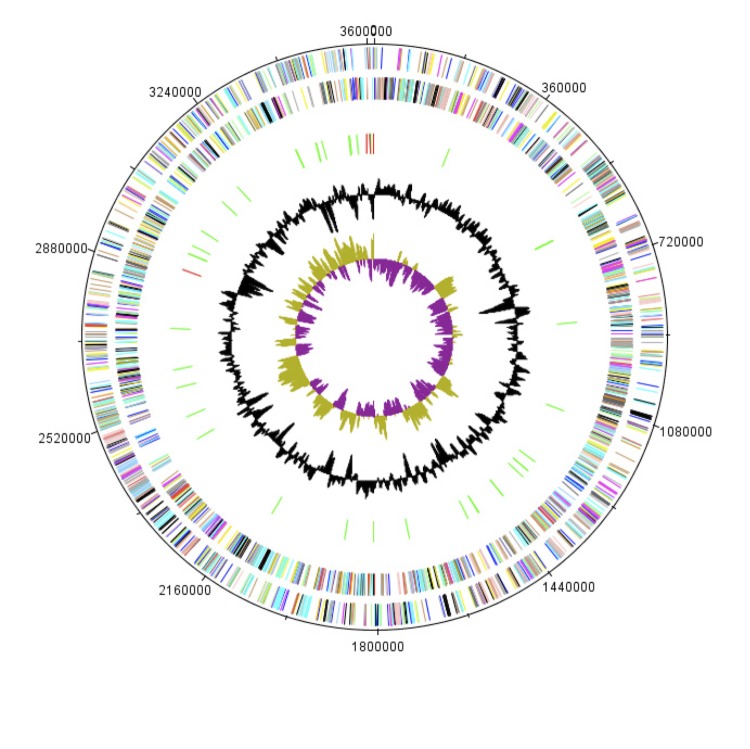
Graphical circular map of the chromosome. From the outside in: open reading frames oriented in the forward (colored by COG categories) direction, open reading frames oriented in the reverse (colored by COG categories) direction, RNA operon (red), and tRNAs (green), GC content plot, and GC skew (purple: negative values, olive: positive values).

The genome is 3,613,503 bp long (1 chromosome, but no plasmid) with a 51.95% G+C content (Figure 6 and Table 4). Of the 3,515 predicted genes, 3,462 were protein-coding genes and 53 were RNAs, including 4 rRNAs. A total of 2,193 genes (62.38%) were assigned a putative function and 81 genes were identified as ORFans (2.3%). The properties and statistics of the genome are summarized in Tables 4 and 5. The distribution of genes into COG functional categories is presented in Table 5.

**Table 4:** Nucleotide content and gene count levels of the genome

**Attribute**	**Value**	% of total^a^
Genome size (bp)	3,613,503	
DNA G+C content (bp)	1,877,214	51.95
DNA Coding region (bp)	3,152,277	87.23
Number of replicons	1	
Extra chromosomal element	0	
Total genes	3,515	100
RNA genes	53	1.51
Protein-coding genes	3,462	98.49
Genes with function prediction	2,193	87.19
Genes assigned to COGs	2,515	71.55
Genes with peptide signals	135	3.84
Genes with transmembrane helices	887	25.23

^a^ The total is based on either the size of the genome in base pairs or the total number of protein-coding genes in the annotated genome

**Table 5:** Number of genes associated with the 25 general COG functional categories

**Code**	**Value**	**% age**^a^	**Description**
J	154	4.45	Translation
A	0	0	RNA processing and modification
K	296	8.55	Transcription
L	138	3.98	Replication, recombination and repair
B	1	0.03	Chromatin structure and dynamics
D	24	0.69	Cell cycle control, mitosis and meiosis
Y	0	0	Nuclear structure
V	73	2.11	Defense mechanisms
T	156	4.5	Signal transduction mechanisms
M	116	3.35	Cell wall/membrane biogenesis
N	62	1.79	Cell motility
Z	0	0	Cytoskeleton
W	0	0	Extracellular structures
U	48	1.38	Intracellular trafficking and secretion
O	66	1.9	Posttranslational modification, protein turnover, chaperones
C	154	4.45	Energy production and conversion
G	237	6.84	Carbohydrate transport and metabolism
E	328	9.47	Amino acid transport and metabolism
F	56	1.61	Nucleotide transport and metabolism
H	92	2.66	Coenzyme transport and metabolism
I	85	2.45	Lipid transport and metabolism
P	164	4.74	Inorganic ion transport and metabolism
Q	53	1.53	Secondary metabolites biosynthesis, transport and catabolism
R	346	10	General function prediction only
S	195	5.63	Function unknown
-	947	27.35	Not in COGs

^a^ The total is based on the total number of protein-coding genes in the annotated genome

## Genome comparison with other *Clostridium* genomes

We compared the genomes of *C. jeddahense* JCD^T^, *C. sporosphaeroides* DSM 1294, *C. leptum* DSM 753, *C. beijerincki* NCIMB 8052, *C. cellulolyticum* H10, *C. difficile* B1, *C. senegalense* DSM 25507, *C. dakarense* DSM 27086 (Table 6A).

**Table 6A:** Genomic comparison of *C. jeddahense* with 7 other *Clostridium* species^†^.

**Species**	**Strain**	**Genome accession** **number**	**Genome size (Mb)**	**G+C content**
*C. jeddahense*	JCD^T^	CBYL00000000	3.61	51.95
*C. sporosphaeroides*	DSM 1294	ARTA01000000	3.17	53.5
*C. cellulolyticum*	H10	NC_011898	4.07	37.4
*C. dakarense*	DSM 27086	CBTZ010000000	3.73	27.98
*C. difficile*	B1	NC_017179	4.46	28.4
*C. leptum*	DSM 753	ABCB02000000	3.27	50.2
*C. senegalense*	DSM 25507	CAEV01000001	3.89	26.8
*C. beijerincki*	NCIMB 8052	NC_009617	6.0	29.0

^†^ A: Species, Strain, GenBank accession number, genome size and G+C content of all compared genomes

The draft genome of *C. jeddahense* (3.61 Mb) is larger than *C. sporosphaeroides* and *C. leptum* (3.17 and 3.27 Mb respectively) but smaller than *C. beijerincki, C. cellulolyticum, C. difficile, C. senegalense* and *C. dakarense* (6.0, 4.07, 4.46, 3.89, 3.73 Mb respectively). It exhibits a higher G+C content than all other compared genome except *C. sporosphaeroides* (53.5%). *C. jeddahense* has a higher gene content (3,462) than *C. sporosphaeroides, C. difficile* (2,951 and 3,390 respectively) but smaller than *C. leptum, C. beijerincki, C. cellulolyticum, C. senegalense* and *C. dakarense* (3,591, 3,818, 3,923, 3,704, 5,020 respectively). *C. jeddahense* shared 1,573, 876, 816, 847, 1,030, 770 and 1,044 orthologous genes with *C. sporosphaeroides, C. cellulolyticum, C. dakarense, C. difficile, C. leptum, C. senegalense* and *C. beijerincki* respectively.

**Figure 7:**
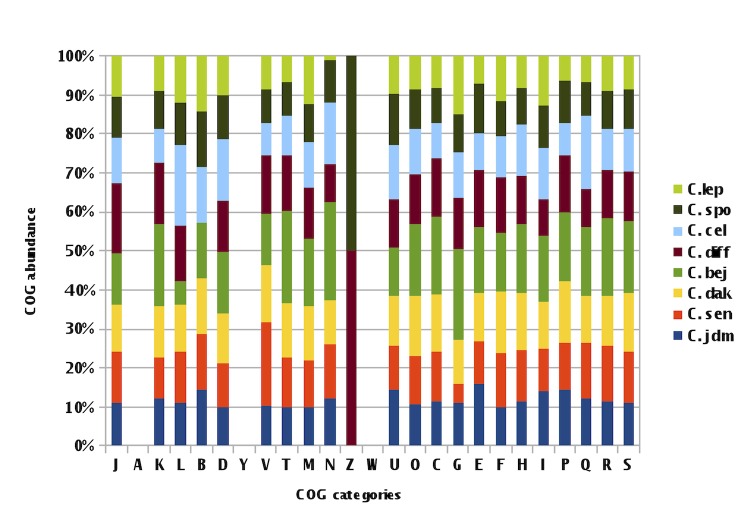
Distribution of predicted genes of *C. jeddahense* and 7 other *Clostridium* species into COG categories. *C.jdm= C. jeddahense*, *C.spo= C. sporosphaeroides*, *C. lep= C. leptum*, *C.bej = C. beijerinckii*, *C. cel = C. cellulolyticum*, *C. diff = C. difficile*, *C. sen = C. senegalense*, *C. dak = C. dakarense*.

When we compared *C. jeddahense* with other species, AGIOS values ranged from 57.52 with *C. senegalense* to 91.97% with *C. sporosphaeroides*. Although the AGIOS value was elevated between *C. jeddahense* and *C. sporosphaeroides*, we believe that the remarkable phenotypic differences, including motility, indole production (Table 2), and protein profile (Figure 7), enable the classification of *C. jeddahense* as a new species.

## Conclusion

On the basis of phenotypic, phylogenetic and genomic analyses (taxono-genomics), we formally propose the creation of *Clostridium jeddahense* sp. nov. that contains strain JCD^T^. This strain was isolated from the fecal flora of an obese 24 year-old Saudian individual living in Jeddah.

### Description of *C. jeddahense* sp. nov.

*Clostridium jeddahense* (jed.dah..en’.se L.gen. neutr. n. combination of Jeddah, the city in Saudi Arabia where the specimen was obtained from an obese Saudian patient sample.) Transparent colonies were 0.2 to 0.3 mm in diameter on blood-enriched agar. *C. jeddahense* is a Gram-positive, obligate anaerobic, endospore-forming bacterium with a mean diameter of 1 µm. Optimal growth on axenic medium was observed at 37°C.

*C. jeddahense* is catalase negative and oxidase negative. Alkaline phosphatase, arginine arylamidase, proline arylamidase, alanine arylamidase, glycine arylamidase, histidine arylamidase, glutamyl glutamic acid arylamidase and serine arylamidase activities were positive. Arginine dihydrolase, α-galactosidase, β-galactosidase, α-glucosidase, β-glucosidase, α-arabinosidase, N-acetyl-β-glucosaminidase, glutamic acid decarboxylase, α-fucosidase, reduction of nitrate, leucyl glycine arylamidase, fermentation of mannose and raffinose, urease, β-galactosidase-6-phosphatase, β-glucuronidase, phenylalanine arylamidase, leucine arylamidase, pyroglutamic acid arylamidase and tyrosine arylamidase activities were negative. Asaccharolytic. Positive for indole. Cells are susceptible to amoxicillin, amoxicillin-clavulanate, imipenem, metronidazole, doxycycline, rifampicin, vancomycin but resistant to ceftriaxone, ciprofloxacin and trimethoprim-sulfamethoxazole.

The G+C content of the genome is 51.95%. The 16S rDNA and genome sequences are deposited in GenBank under accession numbers HG726040 and CBYL00000000, respectively. The type strain is JCD^T^ (= CSUR P693 = DSM 27834).

## References

[1] Tindall BJ Rosselló-Móra R Busse HJ Ludwig W Kämpfer P Notes on the characterization of prokaryote strains for taxonomic purposes. Int J Syst Evol Microbiol 2010; 60:249-266 10.1099/ijs.0.016949-0 19700448

[2] Stackebrandt E Ebers J Taxonomic parameters revisited: tarnished gold standards. Microbiol Today 2006; 33:152-155

[3] Wayne LG Brenner DJ Colwell PR Grimont PAD Kandler O Krichevsky MI Moore LH Moore WEC Murray RGE Stackebrandt E Report of the ad hoc committee on reconciliation of approaches to bacterial systematic. Int J Syst Bacteriol 1987; 37:463-464 10.1099/00207713-37-4-463

[4] Rossello-Mora R. DNA-DNA Reassociation Methods Applied to Microbial Taxonomy and Their Critical Evaluation. In: Stackebrandt E (ed), Molecular Identification, Systematics, and population Structure of Prokaryotes. Springer, Berlin, 2006; p. 23-50.

[5] Welker M Moore ER Applications of whole-cell matrix-assisted laser-desorption/ionization time-of-flight mass spectrometry in systematic microbiology. Syst Appl Microbiol 2011; 34:2-11 10.1016/j.syapm.2010.11.013 21288677

[6] Ramasamy D Mishra AK Lagier JC Padhmanabhan R Rossi-Tamisier M Sentausa E Raoult D Fournier PE A polyphasic strategy incorporating genomic data for the taxonomic description of new bacterial species. Int J Syst Evol Microbiol 2014; (In press). 10.1099/ijs.0.057091-0 24505076

[7] Kokcha S Mishra AK Lagier JC Million M Leroy Q Raoult D Fournier PE Non-contiguous finished genome sequence and description of *Bacillus timonensis* sp. nov. Stand Genomic Sci 2012; 6:346-355 10.4056/sigs.2776064 23408487 PMC3558959

[8] Lagier JC El Karkouri K Nguyen TT Armougom F Raoult D Fournier PE Non-contiguous finished genome sequence and description of *Anaerococcus senegalensis* sp. nov. Stand Genomic Sci 2012; 6:116-125 10.4056/sigs.2415480 22675604 PMC3359877

[9] Mishra AK Gimenez G Lagier JC Robert C Raoult D Fournier PE Non-contiguous finished genome sequence and description of *Alistipes senegalensis* sp. nov. Stand Genomic Sci 2012; 6:304-314 10.4056/sigs.2625821 PMC3558963 23407265

[10] Lagier JC Armougom F Mishra AK Ngyuen TT Raoult D Fournier PE Non-contiguous finished genome sequence and description of *Alistipes timonensis* sp. nov. Stand Genomic Sci 2012; 6:315-324 10.4056/sigs.2685971 23408657 PMC3558960

[11] Mishra AK Lagier JC Robert C Raoult D Fournier PE Non-contiguous finished genome sequence and description of *Clostridium senegalense* sp. nov. Stand Genomic Sci 2012; 6:386-395 23408737 10.4056/sigs.2766062 PMC3558962

[12] Mishra AK Lagier JC Robert C Raoult D Fournier PE Non-contiguous finished genome sequence and description of *Peptoniphilus timonensis* sp. nov. Stand Genomic Sci 2012; 7:1-11 10.4056/sigs.2956294 23449949 PMC3570796

[13] Mishra AK Lagier JC Rivet R Raoult D Fournier PE Non-contiguous finished genome sequence and description of *Paenibacillus senegalensis* sp. nov. Stand Genomic Sci 2012; 7:70-81 10.4056/sigs.3056450 23459006 PMC3577113

[14] Lagier JC Gimenez G Robert C Raoult D Fournier PE Non-contiguous finished genome sequence and description of *Herbaspirillum massiliense* sp. nov. Stand Genomic Sci 2012; 7:200-209 23407294 10.4056/sigs.3086474 PMC3569391

[15] Kokcha S Ramasamy D Lagier JC Robert C Raoult D Fournier PE Non-contiguous finished genome sequence and description of *Brevibacterium senegalense* sp. nov. Stand Genomic Sci 2012; 7:233-245 10.4056/sigs.3256677 23408786 PMC3569389

[16] Ramasamy D Kokcha S Lagier JC N’Guyen TT Raoult D Fournier PE Non-contiguous finished genome sequence and description of *Aeromicrobium massilense* sp. nov. Stand Genomic Sci 2012; 7:246-257 10.4056/sigs.3306717 23408663 PMC3569385

[17] Lagier JC Ramasamy D Rivet R Raoult D Fournier PE Non-contiguous finished genome sequence and description of *Cellulomonas massiliensis* sp. nov. Stand Genomic Sci 2012; 7:258-270 10.4056/sigs.3316719 23408774 PMC3569388

[18] Lagier JC Karkouri K Rivet R Couderc C Raoult D Fournier PE Non contiguous-finished genome sequence and description of *Senegalemassilia anaerobia* gen. nov., sp. nov. Stand Genomic Sci 2013; 7:343-356 10.4056/sigs.3246665 24019984 PMC3764928

[19] Mishra AK Hugon P Nguyen TT Robert C Couderc C Raoult D Fournier PE Non contiguous-finished genome sequence and description of *Peptoniphilus obesi* sp. nov. Stand Genomic Sci 2013; 7:357-369 10.4056/sigs.32766871 24019985 PMC3764929

[20] Mishra AK Lagier JC Nguyen TT Raoult D Fournier PE Non contiguous-finished genome sequence and description of *Peptoniphilus senegalensis* sp. nov. Stand Genomic Sci 2013; 7:357-369 10.4056/sigs.32766871 24019986 PMC3764932

[21] Lagier JC Karkouri K Mishra AK Robert C Raoult D Fournier PE Non contiguous-finished genome sequence and description of *Enterobacter massiliensis* sp. nov. Stand Genomic Sci 2013; 7:399-412 10.4056/sigs.3396830 24019988 PMC3764934

[22] Hugon P Ramasamy D Rivet R Raoult D Fournier PE Non contiguous-finished genome sequence and description of *Alistipes obesi* sp. nov. Stand Genomic Sci 2013; 7:427-439 10.4056/sigs.3336746 24019990 PMC3764931

[23] Hugon P Mishra AK Nguyen TT Raoult D Fournier PE Non-contiguous finished genome sequence and description of *Brevibacillus massiliensis* sp. nov. Stand Genomic Sci 2013; 8:1-14 10.4056/sigs.3466975 23961307 PMC3739172

[24] Mishra AK Hugon P Nguyen TT Raoult D Fournier PE Non contiguous-finished genome sequence and description of *Enorma massiliensis* gen. nov., sp. nov., a new member of the Family Coriobacteriaceae. Stand Genomic Sci 2013; 8:290-305 10.4056/sigs.3426906 23991260 PMC3746427

[25] Ramasamy D Lagier JC Gorlas A Raoult D Fournier PE Non contiguous-finished genome sequence and description of *Bacillus massiliosenegalensis* sp. Nov. Stand Genomic Sci 2013; 8:264-278 10.4056/sigs.3496989 23991258 PMC3746431

[26] Ramasamy D Lagier JC Nguyen TT Raoult D Fournier PE Non contiguous-finished genome sequence and description of *Dielma fastidiosa* gen. nov., sp. nov., a new member of the Family Erysipelotrichaceae. Stand Genomic Sci 2013; 8:336-351 10.4056/sigs.3567059 23991263 PMC3746426

[27] Mishra AK Pfleiderer A Lagier JC Robert C Raoult D Fournier PE Non contiguous-finished genome sequence and description of *Bacillus massilioanorexius* sp. nov. Stand Genomic Sci 2013; 8:465-479 10.4056/sigs.4087826 24501631 PMC3910694

[28] Hugon P Ramasamy D Robert C Couderc C Raoult D Fournier PE Non-contiguous finished genome sequence and description of *Kallipyga massiliensis* gen. nov., sp. nov., a new member of the family *Clostridiales Incertae Sedis XI.* Stand Genomic Sci 2013; 8:500-515 10.4056/sigs.4047997 24501634 PMC3910704

[29] Padhmanabhan R Lagier JC Dangui NPM Michelle C Couderc C Raoult D Fournier PE Non-contiguous finished genome sequence and description of *Megasphaera massiliensis.* Stand Genomic Sci 2013; 8:525-538 10.4056/sigs.4077819 24501636 PMC3910696

[30] Mishra AK Edouard S Dangui NPM Lagier JC Caputo A Blanc-Tailleur C Ravaux I Raoult D Fournier PE Non-contiguous finished genome sequence and description of *Nosocomiicoccus massiliensis* sp. nov. Stand Genomic Sci 2013; 9:205-219 10.4056/sigs.4378121 24501657 PMC3910558

[31] Mishra AK Lagier JC Robert C Raoult D Fournier PE Genome sequence and description of *Timonella senegalensis* gen. nov., sp. nov., a new member of the suborder *Micrococcineae.* Stand Genomic Sci 2013; 8:318-335 10.4056/sigs.3476977 23991262 PMC3746429

[32] Keita MB Diene SM Robert C Raoult D Fournier PE Non contiguous-finished genome sequence and description of *Bacillus massiliogorillae* sp. nov. Stand Genomic Sci 2013; 9:93-105 10.4056/sigs.4388124 24501648 PMC3910557

[33] Mediannikov O El Karkouri K Robert C Fournier PE Raoult D Non contiguous-finished genome sequence and description of *Bartonella florenciae* sp. nov. Stand Genomic Sci 2013; 9:185-196 10.4056/sigs.4358060 24501655 PMC3910550

[34] Lo CI Mishra AK Padhmanabhan R Samb Ba B Gassama Sow A Robert C Couderc C Faye N Raoult D Fournier PE Fenollar F Non contiguous-finished genome sequence and description of *Clostridium dakarense* sp. nov. Stand Genomic Sci 2013; 9:14-27 10.4056/sigs.4097825 24501642 PMC3910555

[35] Mishra AK Hugon P Robert C Raoult D Fournier PE Non contiguous-finished genome sequence and description of *Peptoniphilus grossensis* sp. nov. Stand Genomic Sci 2012; 7:320-330 23408485 10.4056/sigs.3076460 PMC3569384

[36] Mediannikov O El Karkouri K Diatta G Robert C Fournier PE Raoult D Non contiguous-finished genome sequence and description of *Bartonella senegalensis* sp. nov. Stand Genomic Sci 2013; 8:279-289 10.4056/sigs.3807472 23991259 PMC3746424

[37] Roux V Million M Robert C Magne A Raoult D Non-contiguous finished genome sequence and description of *Oceanobacillus massiliensis* sp. nov. Stand Genomic Sci 2013; •••:9 10.4056/sigs.4267953 PMC4062624 24976893

[38] Wells CL, Wilkins TD. (1996). *Clostridia:* Spore forming Anaerobic Bacilli In: *Baron's Medical Microbiology* (Baron S *et al.*, eds.) (4th ed.). University of Texas Medical Branch. 21413315

[39] Field D Garrity G Gray T Morrison N Selengut J Sterk P Tatusova T Thomson N Allen MJ Angiuoli SV The minimum information about a genome sequence (MIGS) specification. Nat Biotechnol 2008; 26:541-547 10.1038/nbt1360 18464787 PMC2409278

[40] Woese CR Kandler O Wheelis ML Towards a natural system of organisms: proposal for the domains *Archaea, Bacteria*, and *Eucarya.* Proc Natl Acad Sci USA 1990; 87:4576-4579 10.1073/pnas.87.12.4576 2112744 PMC54159

[41] Garrity GM, Holt JG. The Road Map to the Manual. In: Garrity GM, Boone DR, Castenholz RW (eds), Bergey's Manual of Systematic Bacteriology, Second Edition, Volume 1. Springer, New York 2001; 119-169.

[42] Gibbons NE Murray RGE Proposals Concerning the Higher Taxa of Bacteria. Int J Syst Bacteriol 1978; 28:1-6 10.1099/00207713-28-1-1

[43] Murray RGE. The Higher Taxa, or, a Place for Everything...? In: Holt JG (ed), Bergey's Manual of Systematic Bacteriology, First Edition, Volume 1, The Williams and Wilkins Co., Baltimore, 1984, p. 31-34.

[44] List of new names and new combinations previously effectively, but not validly, published. List no. 132. Int J Syst Evol Microbiol 2010; 60:469-472 10.1099/ijs.0.022855-0 20458120

[45] Rainey FA. Class II. Clostridia class nov. In: De Vos P, Garrity G, Jones D, Krieg NR, Ludwig W, Rainey FA, Schleifer KH, Whitman WB (eds), Bergey's Manual of Systematic Bacteriology, Second Edition, Volume 3, Springer-Verlag, New York, 2009, p. 736.

[46] Skerman VBD Sneath PHA Approved list of bacterial names. Int J Syst Bact 1980; 30:225-420 10.1099/00207713-30-1-225

[47] Prevot AR. Dictionnaire des bactéries pathogens. *In*: Hauduroy P, Ehringer G, Guillot G, Magrou J, Prevot AR, Rosset, Urbain A (*eds*). Paris, Masson, 1953, p.1-692.

[48] Pribram E. Klassification der Schizomyceten. Klassifikation der Schizomyceten (Bakterien), Franz Deuticke, Leipzig, 1933, p. 1-143.

[49] Prazmowski A. "Untersuchung über die Entwickelungsgeschichte und Fermentwirking einiger Bakterien-Arten." Ph.D. Dissertation, University of Leipzig, Germany, 1880, p. 366-371.

[50] Smith LDS, Hobbs G. Genus III. Clostridium Prazmowski 1880, 23. In: Buchanan RE, Gibbons NE (eds), Bergey's Manual of Determinative Bacteriology, Eighth Edition, The Williams and Wilkins Co., Baltimore, 1974, p. 551-572.

[51] Ashburner M Ball CA Blake JA Botstein D Butler H Cherry JM Davis AP Dolinski K Dwight SS Eppig JT Gene ontology: tool for the unification of biology. The Gene Ontology Consortium. Nat Genet 2000; 25:25-29 10.1038/75556 10802651 PMC3037419

[52] 16S Yourself database. http://www.mediterranee-infection.com/article.php?larub=152&titre=16s-yourself

[53] Logan NA, De Vos P. Genus I. Clostiridum Prazmowski 1880. In: (Eds?) De Vos P, Garrity D, Jones D, Krieg NR, Ludwig W, Rainey FA, Schleifer KH, Whitman WB. Bergey’a manual of Systematic Bacteriology. Volume 3: The Firmicutes, Springer, 738-828.

[54] Seng P Drancourt M Gouriet F La Scola B Fournier PE Rolain JM Raoult D Ongoing revolution in bacteriology: routine identification of bacteria by matrix-assisted laser desorption ionization time-of-flight mass spectrometry. Clin Infect Dis 2009; 49:543-551 10.1086/600885 19583519

[55] Lagier JC Armougom F Million M Hugon P Pagnier I Robert C Bittar F Fournous G Gimenez G Maraninchi M Microbial culturomics: paradigm shift in the human gut microbiome study. Clin Microbiol Infect 2012; 18:1185-1193 23033984 10.1111/1469-0691.12023

[56] Prodigal. http://prodigal.ornl.gov/

[57] Benson DA Karsch-Mizrachi I Clark K Lipman DJ Ostell J Sayers EW GenBank. Nucleic Acids Res 2012; 40:D48-D53 10.1093/nar/gkr1202 22144687 PMC3245039

[58] Lowe TM Eddy SR tRNAscan-SE: a program for improved detection of transfer RNA genes in genomic sequence. Nucleic Acids Res 1997; 25:955-964 10.1093/nar/25.5.0955 9023104 PMC146525

[59] Lagesen K Hallin P Rodland EA Staerfeldt HH Rognes T Ussery DW RNAmmer: consistent and rapid annotation of ribosomal RNA genes. Nucleic Acids Res 2007; 35:3100-3108 10.1093/nar/gkm160 17452365 PMC1888812

[60] Bendtsen JD Nielsen H von Heijne G Brunak S Improved prediction of signal peptides: SignalP 3.0. J Mol Biol 2004; 340:783-795 10.1016/j.jmb.2004.05.028 15223320

[61] Krogh A Larsson B von Heijne G Sonnhammer EL Predicting transmembrane protein topology with a hidden Markov model: application to complete genomes. J Mol Biol 2001; 305:567-580 10.1006/jmbi.2000.4315 11152613

[62] Zhou Y Liang Y Lynch KH Dennis JJ Wishart DS PHAST: a fast phage search tool. Nucleic Acids Res 2011; 39:W347-W352 10.1093/nar/gkr485 21672955 PMC3125810

[63] Aziz RK Bartels D Best AA DeJongh M Disz T Edwards RA Formsma K Gerdes S Glass EM Kubal M The RAST Server: rapid annotations using subsystems technology. BMC Genomics 2008; 9:75 10.1186/1471-2164-9-75 18261238 PMC2265698

[64] Rutherford K Parkhill J Crook J Horsnell T Rice P Rajandream MA Barrell B Artemis: sequence visualization and annotation. Bioinformatics 2000; 16:944-945 10.1093/bioinformatics/16.10.944 11120685

[65] Carver T Thomson N Bleasby A Berriman M Parkhill J DNAPlotter: circular and linear interactive genome visualization. Bioinformatics 2009; 25:119-120 10.1093/bioinformatics/btn578 18990721 PMC2612626

[66] Darling AC Mau B Blattner FR Perna NT Mauve: multiple alignment of conserved genomic sequence with rearrangements. Genome Res 2004; 14:1394-1403 10.1101/gr.2289704 15231754 PMC442156

[67] Lechner M Findeib S Steiner L Marz M Stadler PF Prohaska SJ Proteinortho: Detection of (Co-)orthologs in large-scale analysis. BMC Bioinformatics 2011; 12:124 10.1186/1471-2105-12-124 21526987 PMC3114741

